# Suppression of External NADPH Dehydrogenase—NDB1 in *Arabidopsis thaliana* Confers Improved Tolerance to Ammonium Toxicity via Efficient Glutathione/Redox Metabolism

**DOI:** 10.3390/ijms19051412

**Published:** 2018-05-09

**Authors:** Anna Podgórska, Monika Ostaszewska-Bugajska, Klaudia Borysiuk, Agata Tarnowska, Monika Jakubiak, Maria Burian, Allan G. Rasmusson, Bożena Szal

**Affiliations:** 1 Institute of Experimental Plant Biology and Biotechnology, Faculty of Biology, University of Warsaw, I. Miecznikowa 1, 02-096 Warsaw, Poland; m.ostaszewska@biol.uw.edu.pl (M.O.-B.); k.borysiuk@biol.uw.edu.pl (K.B.); atarnowska@biol.uw.edu.pl (A.T.); monika.jakubiak@student.uw.edu.pl (M.J.); mburian@biol.uw.edu.pl (M.B.); 2 Department of Biology, Lund University, Sölvegatan 35B, SE-223 62 Lund, Sweden; allan.rasmusson@biol.lu.se

**Keywords:** ammonium toxicity, external type II NADPH dehydrogenase, glutathione metabolism, reactive oxygen species, redox homeostasis

## Abstract

Environmental stresses, including ammonium (NH_4_^+^) nourishment, can damage key mitochondrial components through the production of surplus reactive oxygen species (ROS) in the mitochondrial electron transport chain. However, alternative electron pathways are significant for efficient reductant dissipation in mitochondria during ammonium nutrition. The aim of this study was to define the role of external NADPH-dehydrogenase (NDB1) during oxidative metabolism of NH_4_^+^-fed plants. Most plant species grown with NH_4_^+^ as the sole nitrogen source experience a condition known as “ammonium toxicity syndrome”. Surprisingly, transgenic *Arabidopsis thaliana* plants suppressing *NDB1* were more resistant to NH_4_^+^ treatment. The *NDB1* knock-down line was characterized by milder oxidative stress symptoms in plant tissues when supplied with NH_4_^+^. Mitochondrial ROS accumulation, in particular, was attenuated in the *NDB1* knock-down plants during NH_4_^+^ treatment. Enhanced antioxidant defense, primarily concerning the glutathione pool, may prevent ROS accumulation in NH_4_^+^-grown *NDB1*-suppressing plants. We found that induction of glutathione peroxidase-like enzymes and peroxiredoxins in the *NDB1*-surpressing line contributed to lower ammonium-toxicity stress. The major conclusion of this study was that NDB1 suppression in plants confers tolerance to changes in redox homeostasis that occur in response to prolonged ammonium nutrition, causing cross tolerance among plants.

## 1. Introduction

Cellular oxidation-reduction status functions as an integrator of subcellular metabolism and responds to signals from the external environment [1,2,3]. NADP(H) and NAD(H) mediate the flow of reductive power between cellular processes [4], and NAD(P)H can be oxidized by the mitochondrial electron transport chain (mtETC), located in the inner mitochondrial membrane. In plants, mtETC is composed of four multi-subunit complexes: complex I (NADH dehydrogenase), complex II (succinate dehydrogenase), complex III (cytochrome *c* reductase), and complex IV (cytochrome *c* oxidase). Plant mitochondria also possess unique electron routes that can bypass the pathway from complexes I or II to complex IV. These additional electron pathways include external and internal type II dehydrogenases (NDex and NDin, respectively) and the alternative oxidase (AOX) [5,6]. NAD(P)H dehydrogenases are encoded by three gene families (NDA, NDB and NDC). NDex enzymes encoded by *NDB1* utilize NADPH, whereas the enzymes encoded by *NDB2*, *NDB3*, and *NDB4* utilize NADH; NDB1 and NDB2 isoforms are also Ca^2+^-dependent or Ca^2+^-stimulated [6,7,8]. The NDin isoforms NDA1, NDA2, and NDC1 utilize NADH, while the NDC1 isoform may also utilize NADPH. AOX genes belong to two subfamilies, which in *Arabidopsis* are composed of *AOX1a-d* and *AOX2* [9].

The path of electrons from complex I through ubiquinone to complexes III and IV (cytochrome pathway) couples the oxidation of reductants to the reduction of O_2_ to H_2_O. The accompanying translocation of protons builds an electrochemical proton gradient that drives oxidative phosphorylation by ATP synthase (ATPase) to produce energy-rich ATP molecules. In contrast with the cytochrome pathway, whose activity is under adenylate control, the additional “alternative” routes are not linked to ATP synthesis or controlled by adenylates. This is because the AOX, NDin, and NDex pathways dissipate excess reductants without proton motive force generation. This prevents over-reduction of the ubiquinone pool or hyperpolarization of the mitochondrial membrane potential, which could inhibit respiration. Plant mitochondria play a central role as reductant sinks, regulating cellular redox homeostasis and preventing redox poise in cells [4,10]. Although the additional pathways do not contribute to ATP production, their activity is crucial to minimize the production of reactive oxygen species (ROS). Alternative pathway activity is induced when superfluous reductants require oxidation [11,12]. Plant mitochondria can oxidize NAD(P)H from the matrix via complex I or via NDin, and can also oxidize cytosolic NAD(P)H present in the intermembrane space via NDex activity. NDex and NDin have low substrate affinity for NAD(P)H [13] and may be important when excessive reducing power must be oxidized [14]. Whereas, the AOX pathway is important for preventing over-reduction in chloroplasts and to balance redox partitioning during photosynthesis, photorespiration, and respiration [12,15]. Research has indicated the importance of NDin and NDex enzymes in regulating mitochondrial and cytosolic reductant pools. In particular, two *Arabidopsis* RNA interference (RNAi) lines, suppressing the *NDB1* gene for external Ca^2+^-dependent NADPH oxidation by 90%, have highly similar effects on cellular NADP(H) homeostasis and consequentially on growth rate, respiratory metabolism and defense signaling, as shown by metabolome, transcriptome and flux analysis [16].

A disruption in cellular redox homeostasis, which might occur under stress conditions, is deleterious to plant cells because the production of ROS can be dramatically enhanced [17,18]. In plant tissues, ROS are formed by the inevitable leakage of electrons to O_2_ from the electron transport chains of chloroplasts, mtETC, or as a by-product of metabolic pathways, which may be localized in cellular compartments such as peroxisomes. ROS can damage membranes, proteins, and nucleic acids; inhibit enzymes; and even induce programmed cell death in plants [19]. When ROS production is not effectively balanced by scavenging mechanisms, oxidative stress may occur.

To buffer the toxic effects of ROS, plants have developed several antioxidative systems [20]. The major plant low-mass antioxidants include glutathione and ascorbate, which are present in all cellular compartments. The production of ascorbate (the reduced form AsA) in plants is associated with the mtETC, i.e., the last step of synthesis is catalyzed by l-galactono-γ-lactone dehydrogenase, an enzyme attached to complex I [21]. The biosynthesis of glutathione, a tripeptide, requires two enzyme-mediated steps, as characterized in *Arabidopsi*s. The enzymes are glutamate-cysteine ligase (GSH1), which is localized in plastids, and glutathione synthetase (GSH2), which is found in plastids and in the cytosol [22,23].

Glutathione is the most abundant non-protein thiol in cells and its nucleophilic activity is exploited in several stress response pathways to detoxify ROS [23,24]. Reduced glutathione (GSH) can directly reduce all kinds of ROS (^1^O_2_, O_2_·^−^, HO·, H_2_O_2_), while itself being oxidized and forming a disulfide form (GSSG). Moreover, GSH can form disulfides with Cys-residues in proteins (*S*-glutathionylation), protecting them from irreversible oxidation and regulating their activity [25]. Glutathione also has a critical function as an electron donor in other enzymatic detoxification systems. In the ascorbate-glutathione cycle (composed of the enzymes ascorbate peroxidase (APX), dehydroascorbate (DHA) reductase (DHAR), monodehydroascorbate reductase (MDHAR), and glutathione reductase (GR)), APX is ultimately responsible for the decomposition of H_2_O_2_ [26]. The ascorbate-glutathione cycle depends on three redox couples: AsA/DHA, GSH/GSSG, and NAD(P)H/NAD(P), the latter providing the reductant for the former two. In the process of ROS elimination, the network of glutathione peroxidase-like enzymes (GPX), peroxiredoxins (Prx), thioredoxins (TRX) and glutaredoxins (Grx), play an important role [27]. Briefly, GPX and Prx, in their catalytic activity, reduce H_2_O_2_ but also show a strong preference for organic hydroperoxides and peroxinitrite. As a result, GPX and Prx can protect the plant cell not only from ROS but also directly from other stress symptoms such as lipid peroxidation. Further, these enzymes are reduced by TRX in a NADPH- or reduced ferredoxin-dependent manner [28]. Grx, however, can reduce disulfide bonds in protein, a process which is dependent on GSH and that regulates the oxidation state of proteins. Overall, glutathione metabolism requires sulfide group reduction, which is essentially achieved by GR activity using electrons from NADPH. Therefore, GR has been proposed to recycle GSH and to regulate the glutathione redox state in cells [23]. Accordingly, GSH and GSSG form a redox couple that mediate the transfer of reducing equivalents from NADPH to ROS. It is well established that glutathione and ascorbate have a redox potential and are involved in the redox regulation of cells [29,30]. In recent years, the contribution of these low-mass antioxidants to redox-connected retrograde signaling has been identified.

Changes in redox state can be evaluated in plant tissues using different approaches. The role of mitochondria and chloroplasts in maintaining cellular redox balance has been demonstrated in studies in which the activity of the mtETC was altered using inhibitors [31,32]. Mutants are useful tools for investigating the influence of specific proteins in vivo on redox metabolism in plant cells. Another strategy is to investigate stress conditions affecting the intracellular redox balance. The responses of plants to diverse abiotic and biotic stress factors have been analyzed extensively in recent years. In their natural environment, plants must adapt to fluctuations in the availability of nutrients, including nitrogen; this implies the potential existence of a method of sensing mineral availability and executing a rapid metabolic response [2,33]. Plants can utilize two different inorganic nitrogen sources, mostly in the forms of ammonium (NH_4_^+^) or nitrate (NO_3_^−^) [34]. It has been proposed that the application of NH_4_^+^ as the sole source of nitrogen affects redox homeostasis in plant cells [35,36]. When comparing NH_4_^+^ with NO_3_^−^ nutrition, a surplus of reductants may be expected in plant tissues; this is because NO_3_^−^ must first be reduced to NH_4_^+^ in a two-step reduction reaction before its incorporation into amino acids. In the first step, NO_3_^−^ must be reduced to nitrite (using NADH) in a reaction catalyzed by cytosolic nitrate reductase (NR), and then to NH_4_^+^ (using NADPH or reduced ferredoxin) via nitrite reductase (NiR) in the plastids [37,38]. In contrast, when NH_4_^+^ is supplied as the exclusive nitrogen source, thus NO_3_^−^ reduction is omitted, the consumption of reducing equivalents is lower in the cytosol and chloroplasts. In terms of energy economy, NH_4_^+^ seems to represent a better source of nitrogen nutrition for plants, as its assimilation requires less energy than NO_3_^−^ [37,39]. Ammonium nutrition can, however, potentially lead to an accumulation of reductants in these compartments, and consequentially NDex, NDin and AOX enzymes have been observed to be acutely induced by short-term NH_4_^+^ supply [38]. Nevertheless, plants cultured on NH_4_^+^ as the sole nitrogen source exhibit symptoms of toxicity (e.g., growth inhibition), commonly referred to as “ammonium syndrome” [40,41]. We have previously shown that an intracellular redox imbalance during NH_4_^+^ assimilation may lead to oxidative stress in *Arabidopsis* plant tissues [42]. The increased mtETC activity and possible over-reduction of the mtETC in response to NH_4_^+^ nutrition can also induce excessive mitochondrial ROS generation. Plant mitochondria have subsequently been identified as the primary source of ROS during extended NH_4_^+^ nutrition [42,43].

Extensive research has been devoted to the identification of the mechanisms behind the causes of NH_4_^+^ toxicity in plants, as it would be advantageous to minimize the effects of ammonium syndrome as a way to increase crop biomass production [44,45,46]. Mitochondria-associated metabolic reactions such as respiration may potentially increase nitrogen use efficiency [47,48,49]. It can therefore be expected that, for the proper functioning of plants grown on NH_4_^+^ as the sole source of nitrogen, it is important to dissipate excess reducing power arising in the cytosol or chloroplast. Since NDex are the major entry point of cytosolic NAD(P)H not used in metabolic reactions, their function may be primarily important during NH_4_^+^ nutrition. This was also indicated, in our previous study, where the main isoform of additional dehydrogenases elevated under growth on NH_4_^+^ was NDB1 [42].

The aim of the present study was to investigate the role of the Ca^2+^-dependent external NADPH dehydrogenase, NDB1, during NH_4_^+^ nutrition. Transgenic *Arabidopsis* RNA interference lines suppressing NDB1 [16] were utilized in these studies. In the absence of NDex, an over-reduction of the redox-state might represent a burden for plants during NH_4_^+^ nutrition. However, NDB1 knock-downs were surprisingly less sensitive to prolonged NH_4_^+^ nutrition. In this study, we investigated redox/ROS homeostasis in plants grown on different nitrogen sources. It was shown that the improved tolerance to NH_4_^+^ in *NDB1* suppressing plants may be connected to elevated glutathione metabolism, including *S*-glutathionylation of proteins or altered responses of the redox regulatory network.

## 2. Results

**Figure 1:**
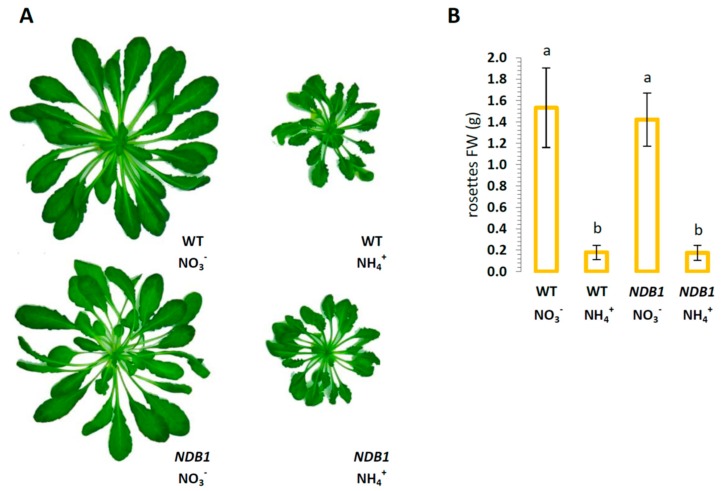
Phenotype of *Arabidopsis thaliana NDB1*-suppressing line 1.5 (*NDB1*) and wild type ecotype Col-0 (WT) after 8 weeks’ growth in hydroponic cultures on 5 mM NO_3_^−^ or 5 mM NH_4_^+^ as the sole nitrogen source. (**A**) Visual appearance of representative plants. All images are in the same scale; (**B**) Fresh weight (FW) of rosettes of WT and *NDB1* knock-down plants on respective nitrogen sources. Bars with different letters are statistically different (*p* < 0.05) by ANOVA.

*NDB1* knock-down plants grown for 8 weeks in hydroponic culture under a short-day photoperiod had a similar rosette size and fresh weight as wild-type (WT) plants grown on the same nitrogen source (Figure 1A,B). Ammonium nutrition caused approximately 90% growth inhibition in both *NDB1* and WT plants (Figure 1B).

### 2.1. Changes in Respiration and Pyridine Nucleotide Status in NDB1 Suppression Plants Cultured on NO_3_^−^ or NH_4_^+^

In NH_4_^+^-grown plants, excess reductants not used in cytosolic reactions can be oxidized by type II dehydrogenases to prevent over-reduction of the mtETC. In general, ammonium nutrition induced higher total respiration (V_t_), cytochrome pathway capacity (V_cyt_), and alternative pathway capacity (V_alt_) in both *NDB1* and WT plants (Table 1). Growth on NH_4_^+^ led to a slightly lower V_t_ in *NDB1* knock-down than in WT plants (Table 1). However, V_cyt_ and V_alt_ were similar between *NDB1* knock-down and WT plants for each nitrogen treatment (Table 1).

**Figure 2:**
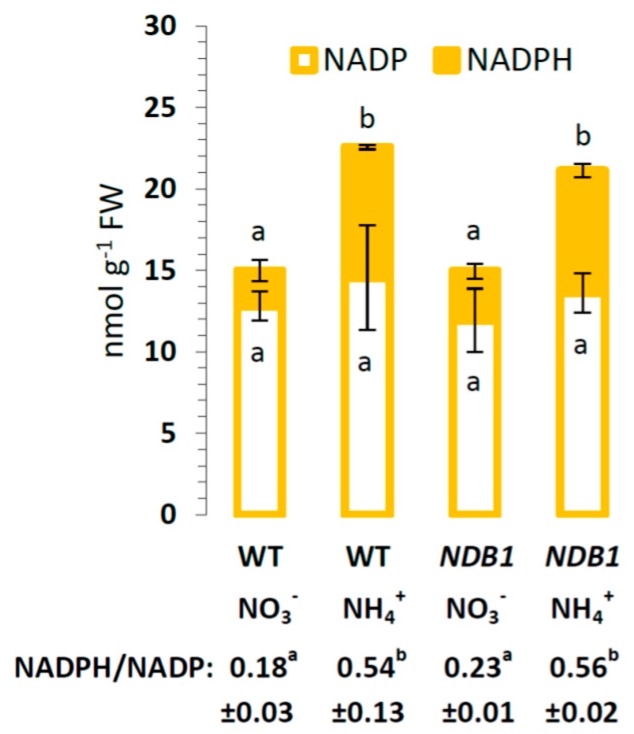
Phosphorylated pyridine nucleotide concentrations in *NDB1*-suppressing and WT plants growing on NH_4_^+^ and NO_3_^−^ (control) as the only source of nitrogen. Bars with different letters are statistically different (*p* < 0.05) by ANOVA.

We next examined how silencing of *NDB1* affects the redox state of pyridine nucleotides. Under nitrate nutrition in *NDB1* knock-down plants, we observed a slightly increased NADPH concentration, similar to that shown in [16]. Overall, however, *NDB1* silencing did not significantly perturb the redox state of NADP(H) in leaves in both growth conditions (Figure 2). The pyridine nucleotide pool was only affected by the applied nitrogen source; ammonium nutrition increased the NADPH content up to four-fold in both genotypes, which resulted in a higher NADPH/NADP ratio (Figure 2).

### 2.2. Influence of NDB1 Suppression on ROS Content and Oxidative Damage in Tissues of Plants Fed NO_3_^−^ or NH_4_^+^

**Figure 3:**
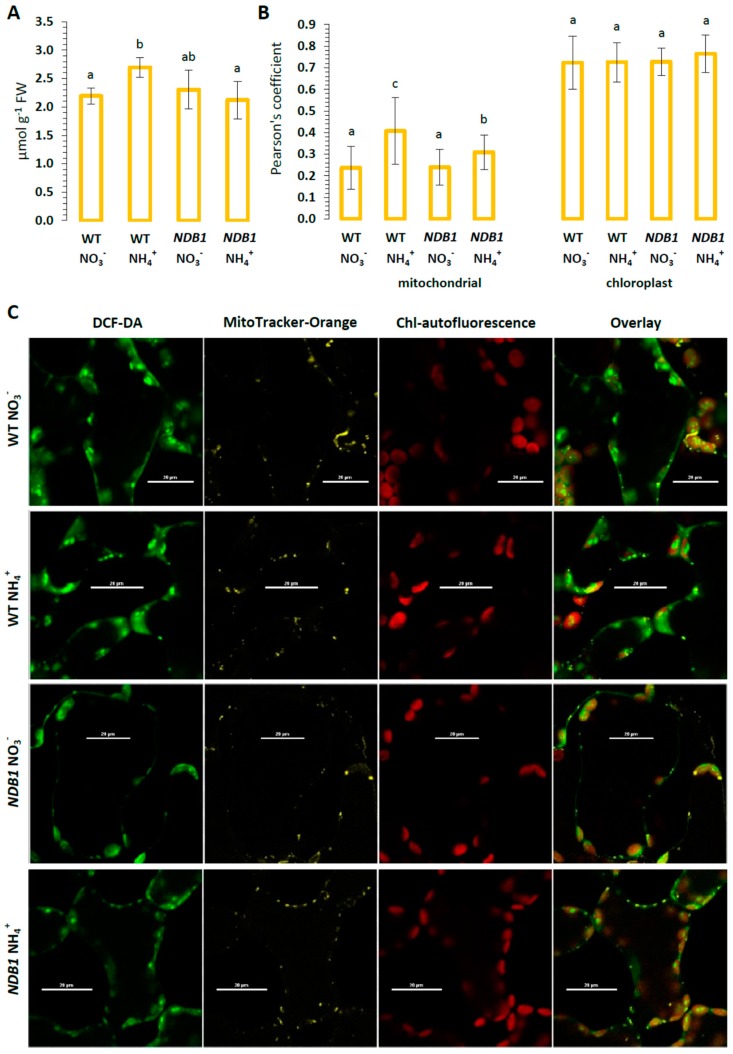
Reactive oxygen species (ROS) in *NDB1*-suppressed and WT plants growing on NH_4_^+^ and NO_3_^−^ (control) as the only source of nitrogen. (**A**) Hydrogen peroxide content; (**B**) sub-cellular ROS distribution in leaf segments. Pearson’s co-localization coefficient shows the proportion of DCF-labelling with mitochondria-dependent fluorescence or chlorophyll autofluorescence. Bars with different letters are statistically different (*p* < 0.05) by ANOVA; (**C**) The mesophyll cells of WT and *NDB1* knock-downs were double-labelled with 2′,7′-dichlorodihydrofluorescein diacetate (DCF-DA, Green) and MitoTracker Orange (reduced CM-H_2_ TMRos, yellow), and chloroplast autofluorescence (far red) was observed; representative images from four independent biological replicates; scale bar = 20 µm.

Altered redox homeostasis in plants may consequently affect ROS production in tissues. In WT plants, the content of H_2_O_2_ was elevated more than 20% in response to NH_4_^+^ nutrition. However, in *NDB1* knock-downs, which had a similar H_2_O_2_ level as WT plants, NH_4_^+^ nutrition did not increase tissue H_2_O_2_ content (Figure 3A). In order to identify the source of ROS in leaf cells, the distribution of H_2_O_2_ in mesophyll tissues was visualized by 2′,7′-dichlorodihydrofluorescein diacetate (DCF-DA) fluorescence. The co-localization of DCF-dependent fluorescence with mitochondria (green/red channels) or with chloroplasts (green/far red channels) (Figure 3C) showed the amount of ROS produced in the respective organelles (Figure 3B). Chloroplasts revealed an obvious fluorescent overlap that co-localized in the DCF-dependent fluorescence, showing a Pearson’s coefficient higher than 0.7 in all analyzed variants (Figure 3B). The detection rate of co-localization between DCF- and mitochondria-dependent fluorescence was the highest in NH_4_^+^-exposed WT cells (Figure 3B), which suggests higher mitochondrial ROS production in these organelles. The defect in NDB1 in the mtETC of knock-down plants did not change the degree of DCF-fluorescence as compared with that of mitochondria of WT plants grown on NO_3_^−^. However, during the NH_4_^+^ growth regime, DCF-staining connected to mitochondria was only slightly increased in the *NDB1* knock-down (Figure 3B).

**Figure 4:**
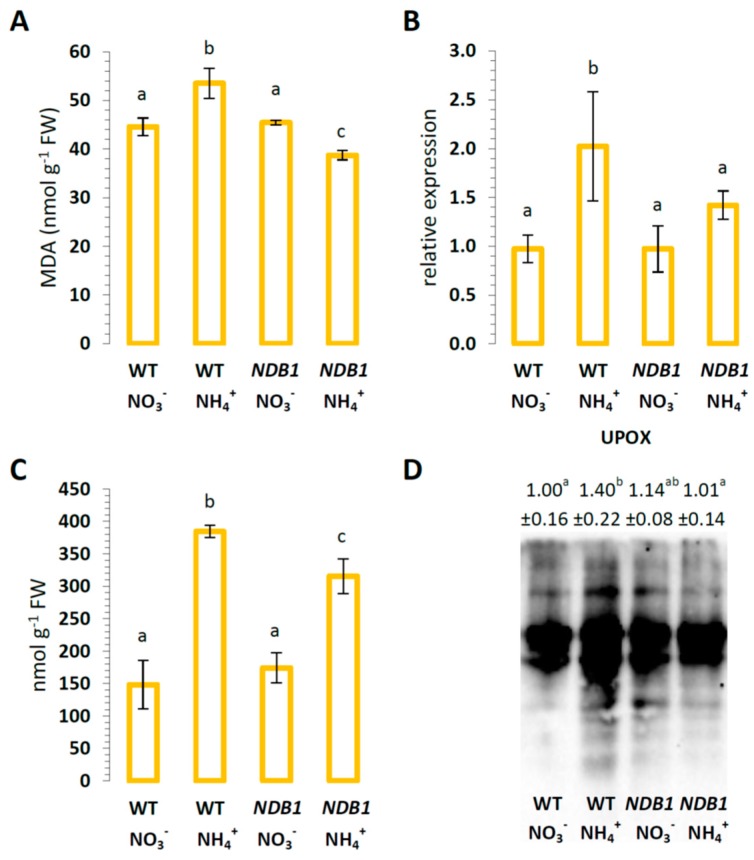
Oxidative stress markers in *NDB1-*suppressed and WT plants grown on NH_4_^+^ and NO_3_^−^ (control) as the only source of nitrogen. (**A**) Lipid peroxidation estimated in leaves as MDA content; (**B**) transcript level for mitochondria-localized protein *UPOX* up-regulated by oxidative stress; (**C**) concentration of carbonylated proteins; and (**D**) immunoblot analysis of protein-bound carbonyls. Bars or bands with different letters are statistically different (*p* < 0.05) by ANOVA.

Since high ROS levels may lead to oxidative modifications of cell components, we estimated malondialdehyde (MDA) content as a marker of peroxidation of membrane lipids, expression of the specific stress response protein up-regulated by oxidative stress (UPOX, [50]), and protein carbonylation level. In WT plants, NH_4_^+^ nutrition, as compared with NO_3_^−^ nutrition, led to a 20% increase in lipid peroxidation (Figure 4A). Lipid peroxidation in *NDB1* knock-down plants grown on NO_3_^−^ was similar to that in WT plants (Figure 4A), but the treatment of *NDB1* knock-down plants with NH_4_^+^ resulted in an 15% lower MDA content in tissues (Figure 4A).

Ammonium nutrition induced a higher *UPOX* transcript level in WT plants grown on NO_3_^−^, but in the *NDB1* knock-down line, its expression was similar to that of the control, independent of the nitrogen source (Figure 4B). The level of protein carbonylation was more than 2.5-times higher in WT plants in response to NH_4_^+^ nutrition (Figure 4C). In *NDB1* knock-down plants, which had a similar extent of lipid oxidation to control plants, when grown on NO_3_^−^, the content of carbonylated proteins was elevated less than two-fold during NH_4_^+^ nutrition. The profile of proteins with carbonyl groups was also traced by immunoblotting. Ammonium nutrition induced a strong increase in protein carbonyls in WT plants, being 40% elevated within the whole protein spectrum (Figure 4D). However, the *NDB1* knock-down plants had an unchanged pattern of carbonylated proteins compared with control plants, and this pattern was not further increased by the NH_4_^+^ treatment.

Overall, our data indicate that unlike WT, *NDB1* knock-down plants do not develop major oxidative stress symptoms in response to ammonium nutrition, an effect that may be attributable to efficient antioxidant defense systems.

### 2.3. Ascorbate Level and Ascorbate-Related Antioxidant Defense in NDB1 Knock-Down Plants in Response to NO_3_^−^ or NH_4_^+^ Nutrition

**Figure 5:**
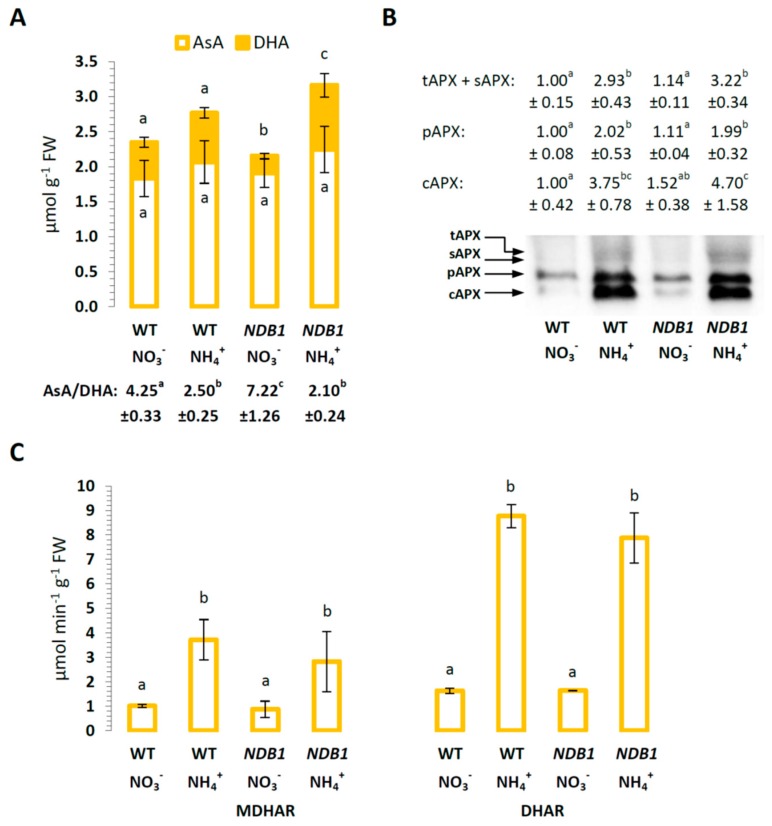
Content of ascorbate and ascorbate-utilizing enzymes in *NDB1-*suppressing and WT plants grown on NH_4_^+^ and NO_3_^−^ (control) as the only source of nitrogen. (**A**) Concentration of reduced (AsA) and oxidized (DHA) ascorbate and derived AsA/DHA ratio; (**B**) ascorbate peroxidase (APX) protein levels. Thylakoid (tAPX, 38 kDa), stromal (sAPX, 33 kDa); peroxisomal (pAPX, 31 kDa), and cytoplasmic (cAPX, 25 kDa) forms of ascorbate peroxidases; (**C**) monodehydroascorbate reductase (MDHAR) and dehydroascorbate reductase (DHAR) activities. Bars or bands with different letters are statistically different (*p* < 0.05) by ANOVA.

Ascorbate is a major low-mass antioxidant. The content of AsA was stable in WT and *NDB1* knock-down plants during growth on different nitrogen sources (Figure 5A). However, the DHA content decreased in the *NDB1* knock-down line during NO_3_^−^ growth compared with WT, which resulted in an elevated AsA/DHA redox state (Figure 5A). In contrast, during growth on NH_4_^+^, an increase in DHA content was detected in the *NDB1* knock-downs compared with WT, but the AsA/DHA ratio remained similar to that of WT plants grown on NH_4_^+^ (Figure 5A). All APX isoforms had similar protein levels in *NDB1* knock-downs compared with WT plants and showed high accumulation under ammonium nutrition in both genotypes (Figure 5B). The same trend was observed for MDHAR and DHAR activities, which were similar between *NDB1* knock-downs and WT plants and were highly induced under ammonium nutrition (Figure 5C).

### 2.4. Glutathione Metabolism in NDB1 Knock-Down Plants during NO_3_^−^ or NH_4_^+^ Growth

**Figure 6:**
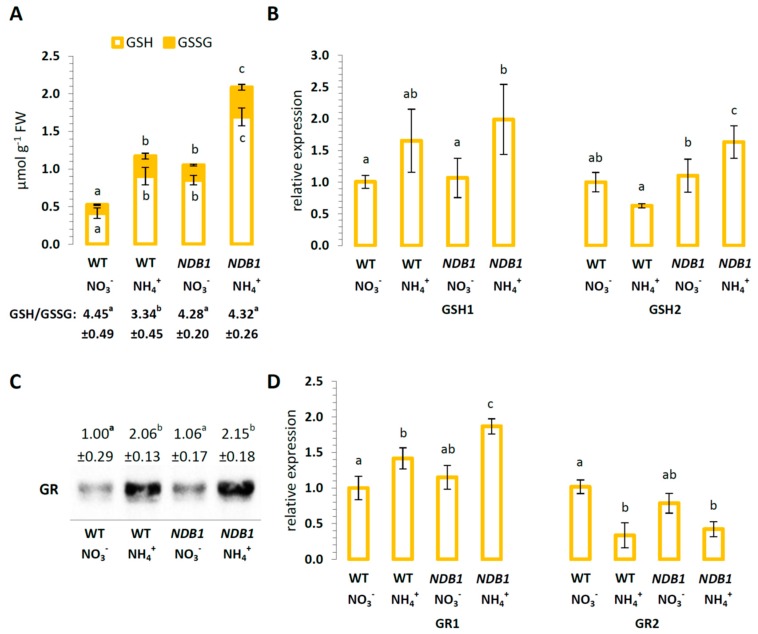
Glutathione contents and glutathione metabolism-related enzymes in *NDB1-*suppressing and WT plants growing on NH_4_^+^ and NO_3_^−^ (control) as the only source of nitrogen. (**A**) Concentration of reduced (GSH) and oxidized (GSSG) glutathione and derived GSH/GSSG ratio; (**B**) transcript levels for *GSH1* and *GSH2*; (**C**) GR protein levels; (**D**) transcript levels for GR isoforms, *GR1* and *GR2.* Bars or bands with different letters are statistically different (*p* < 0.05) by ANOVA.

Ammonium nutrition in WT plants resulted in higher levels of GSH and GSSG and a more oxidized GSH/GSSG ratio compared with NO_3_^−^-grown plants. During NO_3_^−^ nutrition, the *NDB1* knock-down line also showed increased GSH and GSSG content compared with WT plants and the GSH/GSSG ratio was similar in both genotypes (Figure 6A). When grown on NH_4_^+^, the *NDB1* knock-downs had even higher glutathione levels, showing an increase of 90% in the content of GSH and 50% in GSSG (Figure 6A). However, the GSH/GSSG ratio was unchanged compared with the NO_3_^−^ nutrition of *NDB1* knock-down plants. As glutathione is considered the major cellular redox buffer, we can conclude from the redox state of the glutathione pool that *NDB1* knock-down plants are not adversely affected by modifications in their cellular redox state.

The transcript level of both genes from the glutathione biosynthetic pathway, *GSH1* and *GSH2*, was similar between different nitrogen treatments in WT plants, but in *NDB1* knock-down plants, ammonium nutrition stimulated *GSH1* and *GSH2* expression (Figure 6B), leading to a significantly higher glutathione pool (Figure 6A).

A high rate of glutathione reduction in *NDB1* knock-down plants could be explained by the up-regulation of glutathione reductase. We therefore analyzed GR protein levels. Densitometric analysis revealed that GR levels were similar between *NDB1* knock-downs and WT plants grown under control conditions (Figure 6C). Ammonium nutrition resulted in a two-fold increase in GR protein in both WT and *NDB1* knock-down plants (Figure 6C). To further elucidate the involvement of GR in GSH reduction in NH_4_^+^-grown *NDB1* knock-down lines, we analyzed the expression of two GR-encoding genes: *GR1*, which encodes the cytosolic isozyme, and *GR2*, which encodes an isoform dual-targeted to chloroplasts and mitochondria. In *NDB1* knock-down plants grown on NO_3_^−^, the transcript level for both genes was similar to that in WT plants (Figure 6D). Interestingly, *GR1* and *GR2* showed opposite trends of expression between treatments: *GR1* was induced while *GR2* was suppressed by ammonium nutrition in both genotypes.

**Figure 7:**
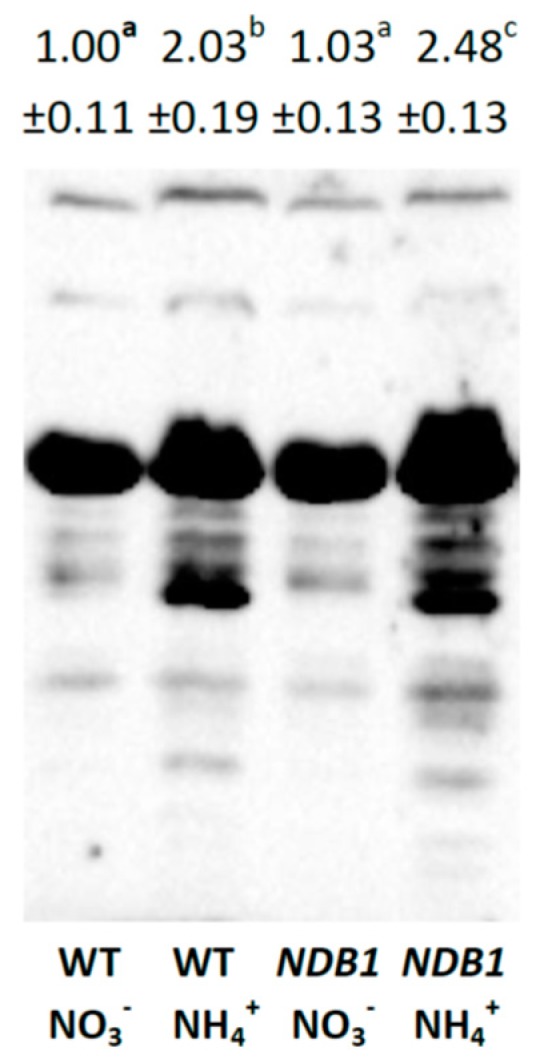
*S*-glutathionylated protein level in *NDB1-*suppressing and WT plants grown on NH_4_^+^ and NO_3_^−^ (control) as the only source of nitrogen. Bands with different letters are statistically different (*p* < 0.05) by ANOVA.

An induction of the glutathione biosynthetic pathway and increase in glutathione content may lead to higher *S*-glutathionylation of proteins. Therefore, we determined the level of this modification in leaf protein extracts by western blotting with anti-GSH antibodies. Densitometry analysis of the entire blot lane revealed that, in WT plants, ammonium nutrition increased the level of protein *S*-glutathionylation compared with NO_3_^−^ nutrition (Figure 7). *NDB1* silencing alone had no effect on *S*-glutathionylation, while in knock-down plants under NH_4_^+^ nutrition, the increase in *S*-glutathionylation of proteins was even more pronounced than in WT plants (Figure 7).

### 2.5. The Effect of NDB1 Suppression on Redox-Related Enzymes in Plants Grown on NO_3_^−^ or NH_4_^+^

**Figure 8:**
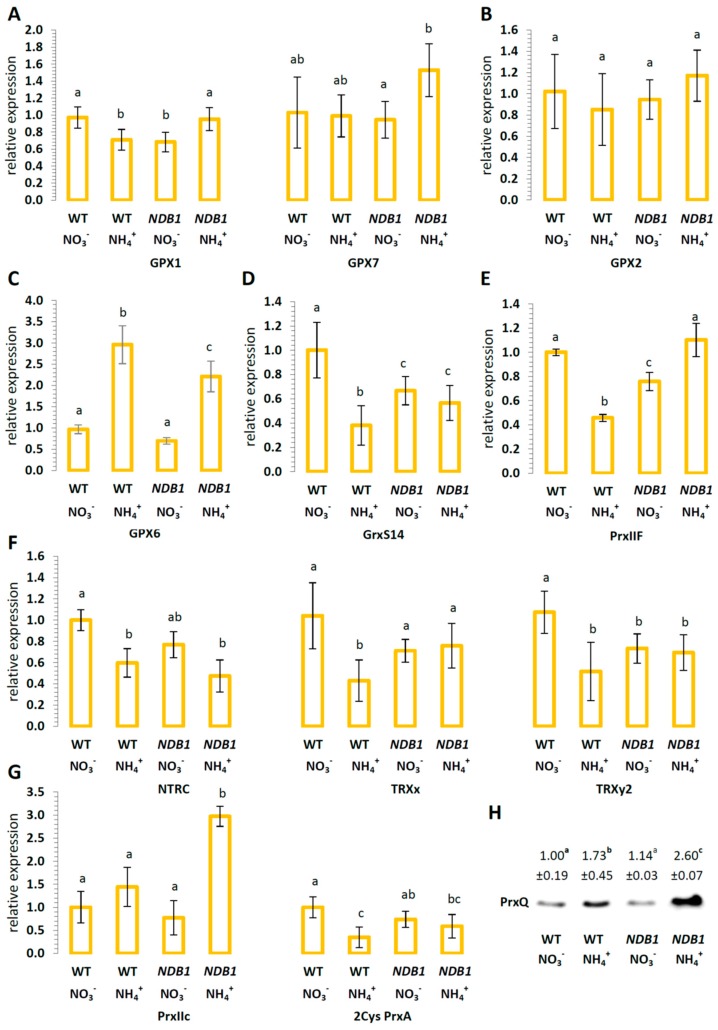
Expression of redox sensors and transmitters in *NDB1-*suppressing and WT plants grown on NH_4_^+^ and NO_3_^−^ (control) as the only source of nitrogen. Transcript levels for (**A**) chloroplast glutathione peroxidase-like (GPX) *GPX1* and *GPX7*; (**B**) cytosolic *GPX2*; (**C**) mitochondrial *GPX6*; (**D**) chloroplast glutaredoxin (Grx) *GrxS14*; (**E**) mitochondrial *PrxIIF*; (**F**) chloroplast NADPH-dependent thioredoxin reductase C (NTRC) and thioredoxins (TRX) *TRXx* and *TRXy2*; (**G**) cytosolic peroxiredoxin (Prx) *PrxIIc* and chloroplast *2Cys PrxA*; and (**H**) PrxQ protein levels. Bars or bands with different letters are statistically different (*p* < 0.05) by ANOVA.

We analyzed the expression profile of different redox related enzymes from the peroxiredoxin, glutathione peroxidase-like, thioredoxin, and glutaredoxin network. In WT plants, chloroplastic isoforms were generally either down-regulated (GPX1, GrxS14, NTRC, TRXx, TRXy2, and 2Cys PrxA) or unchanged (GPX7) in response to NH_4_^+^ nutrition (Figure 8A,D,F,G). The defect in *NDB1* did not alter the expression of most analyzed isoforms in knock-down plants. However, the treatment of *NDB1* knock-down plants with NH_4_^+^ led to an induction of GPX1 and GPX7, while GrxS14, NTRC, TRXx, TRXy2, and 2Cys PrxA remained at a stable level, as in *NDB1* knock-down plants grown on NO_3_^−^ (Figure 8A,D,F,G). The protein level of the chloroplastic peroxidase PrxQ was unchanged between WT and *NDB1* knock-down plants but showed a major increase during NH_4_^+^ nutrition, especially in the *NDB1* knock-down line (Figure 8H). The expression of mitochondrial GPX6 was strongly induced in WT under NH_4_^+^ nutrition, and was also elevated in NH_4_^+^-grown *NDB1* knock-down plants (Figure 8C). Mitochondrial PrxIIF was down-regulated in response to NH_4_^+^ nutrition and in *NDB1* knock-down plants (Figure 8E). Ammonium supply to the *NDB1* knock-down plants increased the expression of mitochondrial PrxIIF (Figure 8E). Furthermore, the expression of cytosolic PrxIIc was induced in *NDB1* knock-down plants when grown on NH_4_^+^ (Figure 8G). The abundance of cytosolic GPX2 (Figure 8B) and GPX8 (Supplementary Materials Figure S2) transcripts was unchanged throughout.

## 3. Discussion

### 3.1. NDB1-Suppressed Line Does Not Show a Growth Phenotype or Over-Reduction under Ammonium Nutrition

*NDB1* knock-down plants do not show a growth phenotype, but have similar growth to WT plants in either NO_3_^−^ or NH_4_^+^-supplied hydroponic growth conditions (Figure 1A,B). Previously, it has been shown that the *NDB1* suppressor line 1.5 (used in this study) grown on soil exhibited decreased biomass [16]. These inconsistent phenotypic responses can be attributed to different growth conditions, e.g., nutrient availability, daylight period (10 h versus 8 h in the present study), or light intensity (80 versus 150 µmol m^−2^ s^−1^).

Under nitrate conditions, the NADP(H) pool showed a reduced trend in the *NDB1* suppressor line (Figure 2). Since ammonium nutrition greatly increases cell redox state [42], plants with impaired *NDB1* were expected to show cellular over-reduction. Surprisingly, under ammonium nutrition, *NDB1* knock-down plants exhibited a similar increase in redox state as WT plants (Figure 2). Maintenance of redox homeostasis is central to plant survival, especially under conditions where elevated redox input from the cytosol to the mtETC is expected. We have previously shown that growth of *A. thaliana* under long-term NH_4_^+^ supply results in up-regulation of *NDB1* expression, among all additional dehydrogenases [42]. Therefore, to compensate for *NDB1* suppression, induction of other type II dehydrogenases might be suspected. However, a lower total leaf respiratory rate in *NDB1* knock-down plants under ammonium nutrition compared with WT (Table 1) strongly suggests that *NDB1* knock-down plants do not fully compensate for the deficiency in NADPH-dependent NDex under these conditions. Noteworthy, the differences in total respiration between genotypes grown on NH_4_^+^ did not result from lower activity of terminal oxidases, since both V_cyt_ and V_alt_ were comparable between WT and *NDB1* knock-down plants (Table 1).

### 3.2. Ammonium Nutrition Causes Less Oxidative Injury in NDB1 Knock-Down Plants

Previously, we have shown that long-term ammonium nutrition results in reductive stress leading to increased ROS production, and consequently resulting in oxidative injury to biomolecules [42]. NDB1 activity seems to be especially important for plant growth under stress conditions, including ammonium nutrition [42], but the opposite was observed, in that the analyzed *NDB1* suppressor plants appeared to be more resistant to NH_4_^+^ treatment. This observation is supported by data on the second examined transgenic line (8.7), similarly, suppressed for *NDB1* [16], which we present in Supplementary Materials Figures S2–S7. All measured stress parameters, including lipid peroxidation, protein carbonylation, and the expression of the stress marker UPOX (Figure 4), indicated milder oxidative stress in tissues of *NDB1* knock-downs than in WT plants grown on NH_4_^+^. This may be the consequence of lower ROS generation, higher capacity of antioxidant systems, or both in the *NDB1* knock-down plants during NH_4_^+^ nutrition as compared with WT plants.

In contrast to what was observed in WT plants [42] under ammonium nutrition, H_2_O_2_ content in the *A. thaliana NDB1* knock-down line was unchanged (Figure 3A). A defect in the mtETC often correlates with alterations in ROS metabolism, mainly concerning complex I mutants [51,52,53,54]. In contrast to complex I dysfunction, genetic modifications of the alternative pathways result in substantially milder phenotypic expression, being mainly affected during stress conditions, as seen in AOX suppressor plants [55,56,57]. Furthermore, in *Nicotiana sylvestris NDB1*-suppressor and *A. thaliana NDB4* knock-down plants, no elevated ROS levels were observed [58,59]. Since long-term ammonium nutrition primarily induces mitochondrial ROS production [42,43], the lower ROS content in NH_4_^+^-treated *NDB1* knock-down plants could be attributable to lower ROS generation in this compartment. Indeed, lower ROS localization was detected in mitochondria during ammonium nutrition of *NDB1* knock-down plants compared with WT (Figure 3B). Under optimal growth conditions, the rate of mitochondrial ROS production is approximately 20 times lower than in chloroplasts [60], but may be more substantial under stress conditions that create a mitochondrial ROS burst and lead to oxidative damage in tissues [61]. An interesting observation is that dysfunction in one alternative dehydrogenase (mtETC component), whose activity is considered a mechanism that greatly reduces ROS production, does not lead to higher mtROS content under specific stress conditions.

### 3.3. Improved Resistance of the NDB1 Knock-Down Line to Ammonium Stress Is Not Related to Ascorbate-Dependent Antioxidant Systems but May Be Attributable to a Glutathione-Dependent System

To determine why the *NDB1* suppressor line shows less oxidative injury under ammonium nutrition, we analyzed changes in antioxidant system functioning between both genotypes under stress conditions. Foyer–Halliwell–Asada cycle function did not appear to be significantly affected by *NDB1* dysfunction, since MDHAR and DHAR activities (Figure 5C), and APX protein level (Figure 5B) were similar in WT and *NDB1* knock-down plants. Furthermore, we did not observe any significant differences in the activity of SOD isoenzymes (Supplementary Materials Figure S1).

We observed that, under nitrate nutrition, the ascorbate redox state in *NDB1* knock-down plants was even further reduced compared with WT plants (Figure 5A). Redox-related metabolic changes in transgenic *N. sylvestris NDB1* sense-suppression plants have previously been shown to correspond with altered ascorbate content [62]. In a metabolomics study, a negative correlation between NADPH level and DHA content was observed [62], which is in line with the decreased DHA levels observed in *NDB1 A. thaliana* knock-down plants in the present study (Figure 5A). Changes in ascorbate redox state may be the result of increased availability of substrate (NADPH) for MDHAR. It was proposed in [62] that changes in ascorbate level may be connected downstream to the NDB1 defect, because ascorbate synthesis takes place in the mtETC. However, it is possible that the changes in ascorbate content/reduction state may instead reflect the chloroplastic pool, since this is the main ascorbate reservoir induced under stress conditions [63]. Although no changes in Foyer–Halliwell–Asada cycle function were detected in tissue extracts between analyzed genotypes (Figure 5B,C), local changes in the activity of enzymes, affecting low-mass antioxidant reduction status in organelles, cannot be excluded.

Clearly, *NDB1* knock-down *A. thaliana* had a marked elevated total glutathione content (Figure 6A). Comparing the rate of oxidation of different ROS forms by ascorbate and glutathione, it appears that glutathione might represent the more potent antioxidant [30] and therefore plays a key role in plant stress tolerance. This effect may be of significant importance since, in *Arabidopsis* cells, the highest glutathione content was found in mitochondria [64], and thus GSH is presumably the primary mitochondrial ROS scavenger. In conclusion, the high glutathione levels in tissues of *NDB1* knock-down plants could primarily be responsible for efficient ROS detoxification in mitochondria (Figure 3B), which was shown to be the predominant ROS source during NH_4_^+^ nutrition.

Elevated total glutathione content may be achieved by the induction of glutathione-synthesizing enzymes located in chloroplasts [65]. Accordingly, NH_4_^+^ grown *NDB1* knock-down plants showed an up-regulation of *GSH1* and *GSH2* (Figure 6B). However, glutathione antioxidant potential depends not only on absolute glutathione concentration, but also on its redox state. The glutathione pool of *NDB1* knock-down plants showed a more reduced redox state than that in WT plants when nourished on NH_4_^+^ (Figure 6A). A high level of glutathione reduction is primarily triggered by the activity of GR, the main regulatory enzyme [66]. Despite a lack of change in GR protein level (Figure 6C), the NH_4_^+^-grown *NDB1* knock-down line showed an induction of cytosolic GR1 on the transcript level (Figure 6D). GR1 has been proposed as the major isoform of GR in plants responsive to stress factors [67] and therefore may be responsible for the reduction state of glutathione in NH_4_^+^-grown *NDB1* knock-down plants.

The glutathione pool is also implicated in the direct protection of proteins against irreversible oxidative injury due to the *S*-glutathionylation process. Cysteine (Cys) residues in proteins are exposed to modification by ROS in a three-step reaction to successively form sulphenic acid (Cys–SOH), sulphinic acid (Cys–SO_2_H), and sulphonic acid (Cys–SO_3_H), which may lead to protein inactivation. Advanced oxidation of Cys residues is irreversible and leads to inevitable protein degradation, although the Cys–SOH group may be protected against further oxidation by reversible *S*-glutathionylation [68]. Glutathionylation typically occurs under oxidative stress conditions and plays an important role in regulation and signaling [69]. Some studies indicate that, under oxidative stress conditions, the activity of enzymes associated with primarily carbon metabolism may be down-regulated due to glutathionylation [69], allowing more effective antioxidant protection of cells. Additionally, reversible *S*-glutathionylation is part of the catalytic cycle of some glutathione-dependent enzymes, including DHAR [23]. Ammonium nutrition results in increased protein glutathionylation in both genotypes, although a higher level of *S*-glutathionylated proteins was observed in *NDB1* knock-down plants (Figure 7). Unfortunately, we cannot determine whether increased *S*-glutathionylation is the result of the protection of Cys-SOH groups in oxidized proteins or whether it is aimed at the regulatory adjustment of metabolism; however, both processes may be responsible for improved tolerance of *NDB1* knock-down plants to NH_4_^+^. The regeneration of native Cys residues in proteins depends on GSH but requires GRXs activity. Interestingly, ammonium treatment results in the specific down-regulation of expression of several members of the GRX gene family [70]. In the present study, we measured only the transcript level of GrxS14, confirming the influence of NH_4_^+^ on Grx expression (Figure 8D). Moreover, we observed that in *NDB1* knock-down plants under nitrate conditions, the transcript level of GrxS14 was lower than in WT plants but was not regulated by NH_4_^+^ (Figure 8D). This observation may suggest that, under ammonium nutrition, Cys residues in *NDB1* knock-downs grown on NH_4_^+^ are more efficiently regenerated to their native forms, meaning that *NDB1*-suppressor lines have more efficient protection against oxidative damage of proteins. However, a reduced GrxS14 transcript level in *NDB1* knock-down plants may indicate that the down-regulation of GRX expression is not specifically in response to NH_4_^+^ nutrition, as was suggested previously [70], but rather to changes in the tissue redox state. The influence of *NDB1* suppression on the expression of other GRX genes and on GRX activity therefore requires further research.

### 3.4. NDB1 Deletion Leads to Changes in the Expression of Genes Involved in the Redox Regulatory Network

Following the observation of different effects of ammonium nutrition on the level of GRX transcripts in WT and *NDB1* knock-down plants (Figure 8D), we analyzed the expression of other genes engaged in redox signal integration. Redox-sensitive proteins were classified into two classes: redox sensors, including Prx and GPX, and redox transmitters, containing a large family of Grxs and TRXs [71]. In general, we can conclude that the expression of all redox-sensitive genes evaluated was regulated differently by NH_4_^+^ in WT and in *NDB1* knock-down plants (Figure 8A,B,D–F), with the exception of GPX2 and GPX8 isoforms, whose expression was unchanged irrespective of growth conditions and genotype (Figure 8B, Supplementary Materials Figure S7C).

Among the four Prx, which are targeted to the plastids, we estimated the expression of 2Cys PrxA and PrxQ (Figure 8G,H). 2Cys PrxA was strongly down-regulated by NH_4_^+^ in WT plants but not in *NDB1* knock-down plants. In contrast, in both genotypes, the protein level of PrxQ was increased in response to NH_4_^+^, but a greater increase was observed in the *NDB1*-suppressing line. Those observations suggest that both Prx can have markedly different roles in leaf cell metabolism, especially under stress conditions. In plants, the highly abundant 2Cys Prx is involved in protecting photosynthetic ETC functioning as reduced amounts of 2Cys Prx were shown to result in decreased levels of D1 protein and of the light-harvesting protein complex associated with photosystem II [72]. 2Cys Prx activity is related to the functioning of intermembrane protein complexes, which enforces that 2Cys Prx (at least in the majority of its conformational states) is associated with thylakoid membranes [73]. By contrast, PrxQ of *Arabidopsis* was shown to be a soluble protein in the lumen of the thylakoid membranes [74]. All Prx are thiol peroxidases and might function in oxidant detoxification [71]. However, according to available data, 2Cys Prx, in addition to a role in photosynthetic energy dissipation [73], and depending on conformational state, may also function as chaperones [75]. The precise function of the 2Cys Prx chaperone activity is not yet known, but it has been suggested that its binding to stromal fructose-1,6-bisphosphatase allows Calvin cycle activity to be maintained under conditions of excessive ROS production [76]. In addition to substrate specificity, both evaluated Prx may differ; 2Cys Prx catalyzes the detoxification of H_2_O_2_, alkyl hydroperoxides, and reactive nitrogen peroxides [77], while PrxQ has been demonstrated as an H_2_O_2_ peroxidase with very low activity toward lipid peroxides [78]. In the context of our results, high light conditions (resulting in higher production of NADPH) have been shown to lead to decreased 2Cys PrxA transcription but increased PrxQ [79]. Furthermore, PrxQ but not 2Cys PrxA was highly induced by the addition of oxidant to *Arabidopsis* tissues [79]. Finally, the expression of PrxQ was shown to be greatly increased in the hypersensitive response to pathogen treatment [80]. On the basis of these observations alongside our results, we may conclude that 2Cys PrxA is down-regulated in response to changes in chloroplastic NADPH and PrxQ is up-regulated in response to H_2_O_2_ and may be an effective organelle-localized antioxidant under stress conditions. However, both Prx are involved in redox sensing and signaling [71].

Interestingly, similarly to PrxQ, the expression of type II Prx was also increased in response to infection by *Melampsora larici-populina* [80] and both Prx were induced by (pro-) oxidants in the same manner [79]. Also, in our study, changes in cytosolic PrxIIC expression were similar to changes in PrxQ (Figure 8G,H). Therefore, we suggest that PrxIIC also protects *NDB1* knock-down plants against oxidative stress under ammonium nutrition.

The regeneration of oxidized Prx requires a supply of electrons from redox transmitters, but target selectivity between chloroplast redox-sensitive proteins exists in this process; NTRC is responsible mainly for 2Cys Prx reduction and PrxQ is regenerated mainly by the proteins of the TRX family [81]. The expression of NTRC and 2Cys Prx has been shown to be strictly co-regulated to maintain proper functioning of the photosynthetic apparatus and the entire chloroplast metabolism [82]. Our results confirm this observation, since the expression of NTRC and 2Cys PrxA is similarly regulated in both genotypes (Figure 8F,G). However, the changes in expression observed in TRXx and TRXy2 do not reflect the altered expression of PrxQ (Figure 8F,G). Furthermore, it should be noted that the Trx-PrxQ system does not show the same specificity as the NTRC-2Cys PrxA system does. 2Cys Prx may also be regenerated by TRX [71]; therefore, the decrease in TRX expression observed in WT plants under ammonium nutrition and in *NDB1* knock-down plants may reflect the down-regulation of a subclass of 2Cys Prx (Figure 8F–H).

PrxIIF is recognized as an important component of the mitochondrial defense system against peroxide stress, and is regenerated in a GSH-dependent manner. The specific substrate for PrxIIF is H_2_O_2_, and the reduction of lipid peroxides is catalyzed through this enzyme approximately 10 times less efficiently [83]. Studies on the response of PrxIIF to stress conditions have classified *PrxIIF* as non-responsive to high light and salt stress [79,84], but it’s up-regulation has been shown as a result of cadmium treatment [83]. Ammonium stress differentially affects PrxIIF expression in WT and *NDB1* knock-down plants (Figure 8E). Lower PrxIIF abundance might have diminished the antioxidant protection of mitochondria of WT plants grown on NH_4_^+^, leading to higher H_2_O_2_ content in those organelles (Figure 3B). Indeed, it was shown previously that under optimal growth conditions, defects in PrxIIF may be compensated for by other compounds of the antioxidant defense system. However, such compensation is insufficient under stress conditions [83], which suggests an essential role for PrxIIF in organellar redox homeostasis.

**Figure 9:**
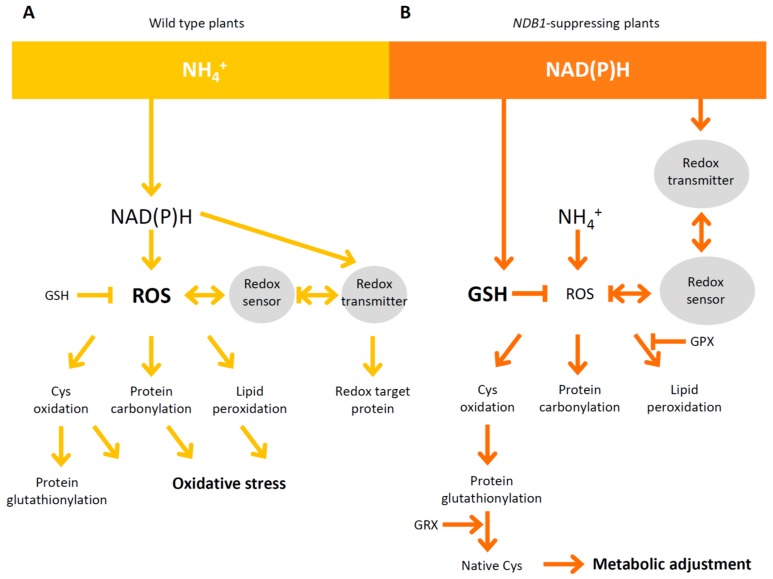
Schematic comparison between redox-related changes occurring in WT and *NDB1* suppressor plants grown on ammonium as the sole nitrogen source. (**A**) Changes in redox homeostasis in WT plants are a direct consequence of ammonium assimilation, and a secondary effect is the induction of redox transmitters/sensors and ROS accumulation leading to protein carbonylation, lipid peroxidation, and oxidation of Cys residues, which causes oxidative stress in NH_4_^+^-grown WT plants; (**B**) *NDB1* knock-down plants have an initial higher redox potential, and can rapidly induce changes in redox-related sensors/transmitters and glutathione content; in these plants, NH_4_^+^ nutrition is an additional factor that does not affect ROS content in tissues. Instead of exhibiting symptoms of oxidative stress symptoms, *NDB1* knock-down plants undergo metabolic adjustment to a high redox input during NH_4_^+^ nutrition. Sharp arrows indicate stimulatory influences, whereas blunt arrows represent inhibitory effects.

Plant GPXs catalyze the reduction of H_2_O_2_ as well as different kinds of lipid peroxides by using TRX as an electron donor. The observed changes in GPX1 and GPX7 expression are relatively small (Figure 8A), with the mitochondrial isoform of GPX showing the only stress-responsive change (Figure 8C). Altogether, among redox sensors, which inactivate ROS, mostly GPX (cytosolic GPX1 and GPX7, and mitochondrial GPX6) and Prx (PrxIIF, PrxIIC, and PrxQ), are up-regulated in *NDB1* knock-down plants under ammonium nutrition. We hypothesize that this up-regulation increases the efficiency of ROS detoxification (including organic peroxide detoxification detected as lower levels of lipid peroxidation, Figure 4A) while enabling plants to better adjust their metabolism to stress conditions (Figure 9).

## 4. Material and Methods

### 4.1. Plant Material and Growth Conditions

*Arabidopsis thaliana* plants of ecotype Columbia-0 (WT) and their derivatives, *NDB1*-suppressed by RNA interference line 1.5 (named *NDB1*) and 8.7 (*NDB1* 8.7, Supplementary Materials Figure S2) [16], were grown hydroponically using an Araponics SA system (Liège, Belgium). Seeds were sown in half-strength [85] basal salt mixture with 1% agar, and after germination (1 week), deionized water in the hydroponic box was replaced with the nutrient solution described in [42], which was constantly aerated and replaced twice a week thereafter. The constant sole source of nitrogen for the plants was 5 mM NO_3_^−^ or 5 mM NH_4_^+^. NO_3_^−^-nourished plants were used as controls. Plants were grown for 8 weeks in a growth chamber under an 8-h photoperiod at 150 µmol m^–2^ s^–1^ photosynthetically active radiation (PAR, daylight and warm white, 1:1; LF-40W, Phillips, Pila, Poland), day/night temperature of 21 °C/18 °C, and approximately 70% relative humidity. All assays were carried out on leaf samples collected in the middle of the light period.

### 4.2. Phenotype Analysis

Ten to 25 plants were randomly selected from two plant cultures (grown on NO_3_^−^ or NH_4_^+^, respectively) and weighed for fresh weight determination (FW). Representative rosettes were photographed.

### 4.3. Respiratory Measurements

Leaves were weighed and pre-incubated before respiratory measurement in an assay solution containing 30 mM 2-(*N*-morpholino)ethanesulfonic acid (MES), pH 6.2 supplemented with 0.2 mM CaCl_2_. Oxygen consumption rates were measured in 3.0 mL assay solution using a Clark-type oxygen electrode (Rank Brothers Ltd., Cambridge, UK) in the dark at a constant temperature of 25 °C [86]. For inhibitor treatments, 10 mM KCN or 20 mM salicylhydroxamic acid (SHAM) in DMSO were used. To measure residual respiration, both inhibitors were added. Total respiration (V_t_), alternative pathway capacity (V_alt_), and cytochrome pathway capacity (V_cyt_) values were determined after the subtraction of the value for residual respiration.

### 4.4. Quantitative RT–PCR Analyses

Total RNA was extracted from 100 mg of leaf tissue using a Syngen Plant RNA Mini kit (Syngen Biotech, Wrocław, Poland). DNase digestion was performed using an RNase-free DNase Set (Qiagen, Hilden, Germany). Complementary DNA was synthesized from 1 μg of total RNA as a template using reverse transcriptase and oligo-dT primers from a Revert Aid H minus first-strand cDNA synthesis kit, according to the manufacturer’s protocol (Thermo Fisher Scientific Inc., Waltham, MA, USA). Thereafter, RNA digestion was performed as described in [87]. Relative transcript abundance was quantified by comparative quantitation analysis. Transcript content was analyzed using iTaq Universal SYBR Green Supermix (Bio-Rad, Hercules, CA, USA) according to the manufacturer’s instructions.

Primer pairs for *GPX1* (At2g25080), *GPX6* (At4g11600), and *GR1* (At3g24170), were as described in [42], and for *UPOX* (At2g21640; [50]), as described previously in [88]. New primers were designed for *GrxS14* (At3g54900), *2Cys PrxA* (At3g11630), *PrxIIC* (At1g65970), *PrxIIF* (At3g06050), *TRXx* (At1g50320), *TRXy2* (At1g43560), *NTRC* (At2g41680), *GR2* (At3g54660), *GSH1* (At4g23100), *GSH2* (At5g27380), *GPX2* (At2g31570), *GPX7* (At4g31870) and *GPX8* (At1g63460) (Supplementary Materials Table S1), with one sequence covering an exon–exon junction if present in the gene sequence. Quantitative RT-PCR reactions were performed using a thermo cycler (CFX Connect™, Bio-Rad) with an annealing temperature of 60 °C. Transcript abundance was normalized to the expression of the reference *PP2A* (At1g13320) gene [89]. Transcript content and qRT-PCR efficiency of target genes were quantified as described in [90]. Results were expressed in relation to those in NO_3_^−^-grown plants (value of 1).

### 4.5. Analysis of Metabolites

A previously reported method was used for the extraction and analysis of NAD(P)(H) [91]. Hydrogen peroxide content was determined according to [92] and quantified with reference to an internal standard (5 nmol H_2_O_2_). Ascorbate was extracted from leaf tissue using 0.1 M hydrochloric acid, and the levels of the reduced (AsA) and oxidized (DHA) forms of ascorbate were assayed according to the [93] method. Glutathione extraction was performed in 5% meta phosphoric acid and GSH was quantified using the enzymatic assay of [94]. Lipid peroxidation was estimated by measuring the secondary oxidation product MDA as described in [95]. Oxidized proteins were labeled with 2,4-dinitrophenylhydrazine (DNPH) and quantified by measuring carbonylated protein derivatives in tissue extracts according to [96]. Soluble protein content in the samples was estimated by the [97] method using bovine serum albumin (BSA) as the standard.

### 4.6. Confocal Fluorescent Imaging of Intracellular ROS

In vivo ROS localization was determined by detecting the fluorescence of DCF and colocalization with mitochondria and chloroplasts using confocal laser scanning microscopy according to the method described in [98], with minor modifications. The upper epidermis was removed from fresh plant leaves, and tissues were cut into small sections (approx. 5 mm). Samples were double-stained with cell-permeant 100 nM MitoTracker Orange (reduced CM-H_2_ TMRos, Molecular Probes, Thermo Fisher Scientific, Waltham, MA, USA) and 20 μM 2′,7′-dichlorodihydrofluorescein diacetate (DCF-DA, Molecular Probes, Thermo Fisher Scientific) under dark incubation for 15 min at room temperature (RT). The leaf pieces were washed in water three times for 5 min and then placed on a glass coverslip. MitoTracker Orange fluorescence was induced with a 561 nm helium-neon laser at emission of 570–620 nm. DCF-fluorescence was measured using the 488 nm line of an argon laser and was monitored at 500–550 nm. Chlorophyll fluorescence was induced with a 633 nm helium-neon laser and detected at 663–738 nm. Images were acquired using a NIKON A1R MP confocal laser scanning system (LSM-510, Carl Zeiss, Oberkochen, Germany) at 60× (numerical aperture 1.2) water immersion objective. Negative control images were obtained by omitting fluorescent probes to remove all signal from tissues except for chlorophyll autofluorescence. For individual cells, Pearson’s colocalization coefficient was calculated between the green/red and green/far red channels using the Nis-Elements 3.22 imaging software (Nikon Co., Tokyo, Japan).

### 4.7. Western Blotting Analyses

To determine protein levels, tissue was homogenized with 2.5 volumes of extraction buffer and 5 µL of the resulting extract was used for GR, 15 µL for APX, 10 µL for PrxQ, and 3 µL for MnSOD. Extracts were subjected to sodium dodecyl sulfate-polyacrylamide gel electrophoresis (SDS-PAGE). The polypeptides were electroblotted onto nitrocellulose membranes using wet transfer (Bio-Rad, Hercules, CA, USA) and probed with anti-PrxQ (diluted 1:5000; Agrisera, Vännäs, Sweden), anti-APX (diluted 1:2000; Agrisera), anti-GR (diluted 1:4000; Agrisera), or anti-MnSOD (diluted 1:1000; Sigma-Aldrich, St. Louis, MO, USA) primary antibodies overnight at 4 °C, followed by anti-rabbit secondary antibodies conjugated to horseradish peroxidase (diluted 1:10,000 for APX and MnSOD, 1:40,000 for GR, and 1:20,000 for PrxQ determination; Bio-Rad). The quantification of protein *S*-glutathionylation was achieved after the detection of glutathione-protein complexes. Tissue extracts (20 µL) were mixed with Laemmli sample buffer containing *N*-ethylmaleimide (NEM, final concentration 5 mM); NEM (2.5 mM) was also present in the blocking buffer to prevent reduction of GSH adducts by thiol-containing proteins [99]. For immunoblotting, anti-GSH (diluted 1:1000; Abcam, Cambridge, UK) antibodies were used, with anti-mouse antibodies (diluted 1:20,000, Bio-Rad) as secondary antibodies. For the determination of carbonylated proteins in electrophoretically separated protein extracts (5 µL), an OxyBlot detection kit (Millipore, Billerica, MA, USA) was used according to the manufacturer’s protocol. Visualization was performed using a chemiluminescence kit (Clarity™ Western ECL, Bio-Rad), and signals were detected using a Chemi-Doc imaging system (Bio-Rad). Specific bands were referred to pre-stained protein markers (Bio-Rad). The densitometry of bands for analyzed proteins or of the whole blot lane for carbonylated and glutathionylated proteins was quantified using Image-Lab 5.2 software (Bio-Rad) after background correction.

### 4.8. Enzyme Activity Assay

For the measurement of ascorbate-glutathione cycle enzymes activity, leaves were homogenized in 50 mM phosphate buffer, pH 7.0, 1 M NaCl, 1% (*w*/*v*) PVP, and 1 mM EDTA (as described in [100]). DHAR was determined according to the method of [101]. MDHAR and GR were assayed as in [102]. Superoxide dismutase (SOD) isoforms were visualized in gel by the method of [103] after electrophoretic separation of protein extracts (6 µL) on 12% polyacrylamide gels.

### 4.9. Statistical Analysis

Results are expressed as the mean value ± standard deviation (SD) of n measurements (*n* = 3–25) taken from at least two independent plant cultures. To analyze the statistical significance of observed differences, a one-way analysis of variance (ANOVA) with Tukey’s post-hoc test at *p*-values ≤0.05 was performed using Statistica 13.1 software (StatSoft, Inc., Tulsa, OK, USA).

## 5. Conclusions

We have shown that the impairment of NDB1 in mitochondrial ETC does not trigger major oxidative stress in plants challenged with NH_4_^+^ treatment. Moreover, high light conditions were not a burden for plants with suppressed *NDB1* [16,62]. On the basis of a microarray study to identify a response overlap, the *NDB1 Arabidopsis thaliana* suppressor line exhibited a similar profile to OsHsfA21 plants, which have been characterized by improved tolerance to stress, including pathogen resistance [16]. It has also been shown that the suppression of another NADH-dependent NDex isoform, NDB4, improved salinity stress tolerance [59]. Altogether, these findings may lead to the assumption that the suppression of NDex and subsequent changes in redox metabolism induce cross tolerance among plants to withstand other stresses. This effect may be attributed to the observation that various stresses produce a similar effect to oxidative stress at the cellular level. An epigenetic response in plants may transmit the acquired stress resistance to their progeny [104,105]. This study shows that improved sensitivity to changes connected with altered redox/ROS status in *NDB1* knock-down plants may be beneficial during stress related to ammonium nutrition.

## Figures and Tables

**Table 1:** Respiratory parameters in *NDB1-*suppressing and WT *Arabidopsis* plants grown on NH_4_^+^ and NO_3_^−^ (control) as the only source of nitrogen. Values are shown for total respiration (V_t_), cytochrome pathway capacity (V_cyt_), and alternative pathway capacity (V_alt_) in dark-adapted leaves. Results with different letters are statistically different (*p* < 0.05) by ANOVA.

	WT NO_3_^−^	WT NH_4_^+^	*NDB1* NO_3_^−^	*NDB1* NH_4_^+^
Oxygen consumption (nmol O_2_ min^−^^1^ g^−^^1^ FW)				
Total respiration (V_t_)	40.54 ^a^ ± 9.93	93.16 ^c^ ± 3.67	41.38 ^a^ ± 5.99	72.92 ^b^ ± 2.96
+SHAM (V_cyt_)	37.31 ^a^ ± 7.65	66.09 ^b^ ± 6.39	36.46 ^a^ ± 10.84	55.45 ^ab^ ± 4.62
+KCN (V_alt_)	5.94 ^a^ ± 3.11	38.98 ^b^ ± 10.58	5.82 ^a^ ± 1.38	46.47 ^b^ ± 2.74

## Supplementary Materials

Supplementary materials can be found at http://www.mdpi.com/1422-0067/19/5/1412/s1.

## Abbreviations

APX	ascorbate peroxidase
AsA	reduced form of ascorbate
DHA	oxidized form of ascorbate (dehydroascorbate)
DHAR	dehydroascorbate reductase
GPX	glutathione peroxidase-like
GR	glutathione reductase
GRX	glutaredoxin
GSH	reduced form of glutathione
GSSG	oxidized form of glutathione (glutathione disulfide)
MDHAR	monodehydroascorbate reductase
NDB1	external NADPH dehydrogenase
NTRC	NAPDH-dependent thioredoxin reductase C
Prx	peroxiredoxin
ROS	reactive oxygen species
SOD	superoxide dismutase
TRX	thioredoxin
UPOX	protein up-regulated by oxidative stress
