# Supplementary material for: Suppression of External NADPH Dehydrogenase—NDB1 in Arabidopsis thaliana Confers Improved Tolerance to Ammonium Toxicity via Efficient Glutathione/Redox Metabolism
Source: Int J Mol Sci. 2018 May 9;19(5):1412. doi: 10.3390/ijms19051412 (PMC5983774; doi:10.3390/ijms19051412)
Supplement: Supplementary file 1 [file ijms-19-01412-s001.pdf]

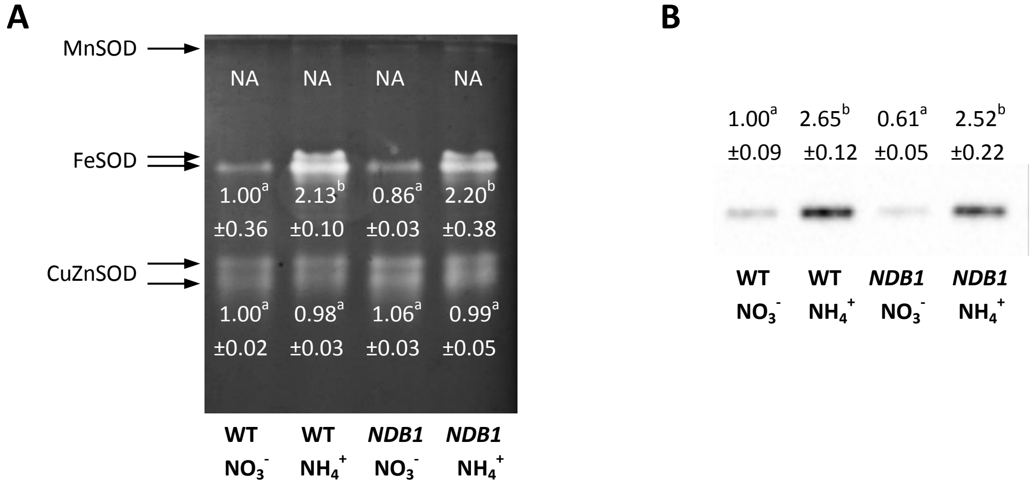

Figure S1. Superoxide dismutase (SOD) in *NDB1*-suppressing line 1.5 (*NDB1*) and wild-type (WT) plants growing on NH_4^+ and NO_3^- (control) as the only source of nitrogen. (A) SOD *in gel* activity and (B) MnSOD levels. Densitometric analysis of SOD activity is described under the respective bands on the blot. NA, not analyzed. Bands with different letters are statistically different ($P < 0.05$) by ANOVA.

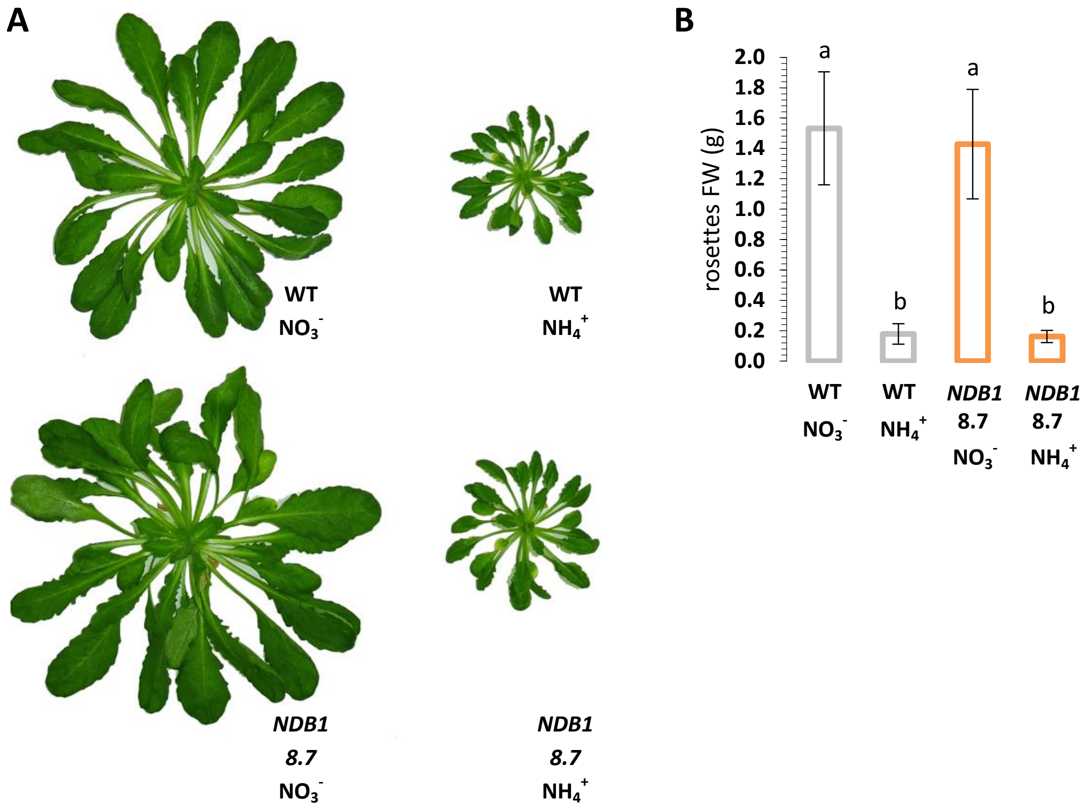

Figure S2. Phenotype of *Arabidopsis thaliana NDB1*-suppressing line 8.7 (*NDB1 8.7*) and WT ecotype Col-0 (WT) after 8 weeks' growth in hydroponic cultures on 5 mM NO_3^- or 5 mM NH_4^+ as the sole nitrogen source. (A) Visual appearance of representative plants. All images are in the same scale. (B) Fresh weight (FW) of rosettes of WT and *NDB1* knock-down line 8.7 on respective nitrogen sources. Bars with different letters are statistically different (P < 0.05) by ANOVA.

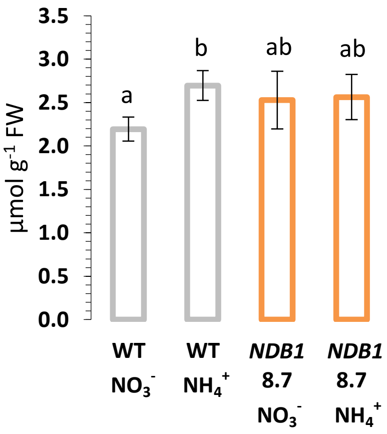

Figure S3. Hydrogen peroxide content of *NDB1*-suppressing line 8.7 and WT plants growing on NH_4^+ and NO_3^- (control) as the only source of nitrogen. Bars with different letters are statistically different (P < 0.05) by ANOVA.

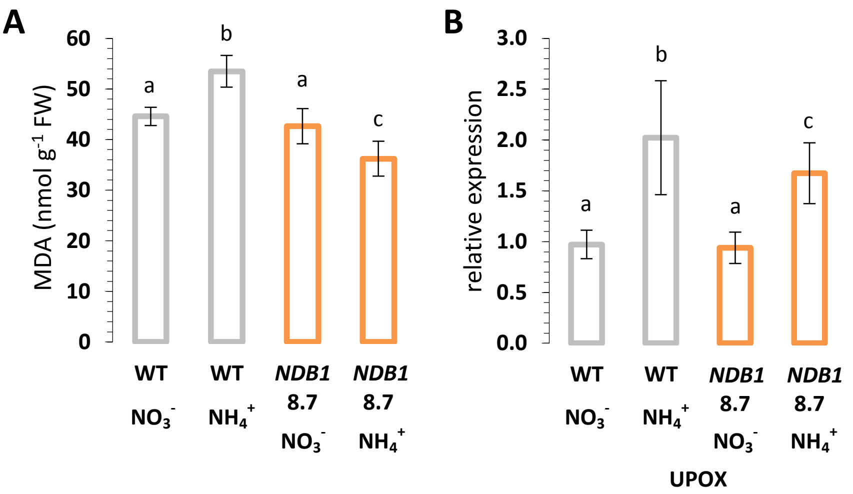

Figure S4. Oxidative stress markers in *NDB1*-suppressing line 8.7 and WT plants growing on NH_4^+ and NO_3^- (control) as the only source of nitrogen. (A) Lipid peroxidation estimated in leaves as MDA content and (B) transcript level for mitochondrial protein *UPOX* up-regulated by oxidative stress. Bars with different letters are statistically different (P < 0.05) by ANOVA.

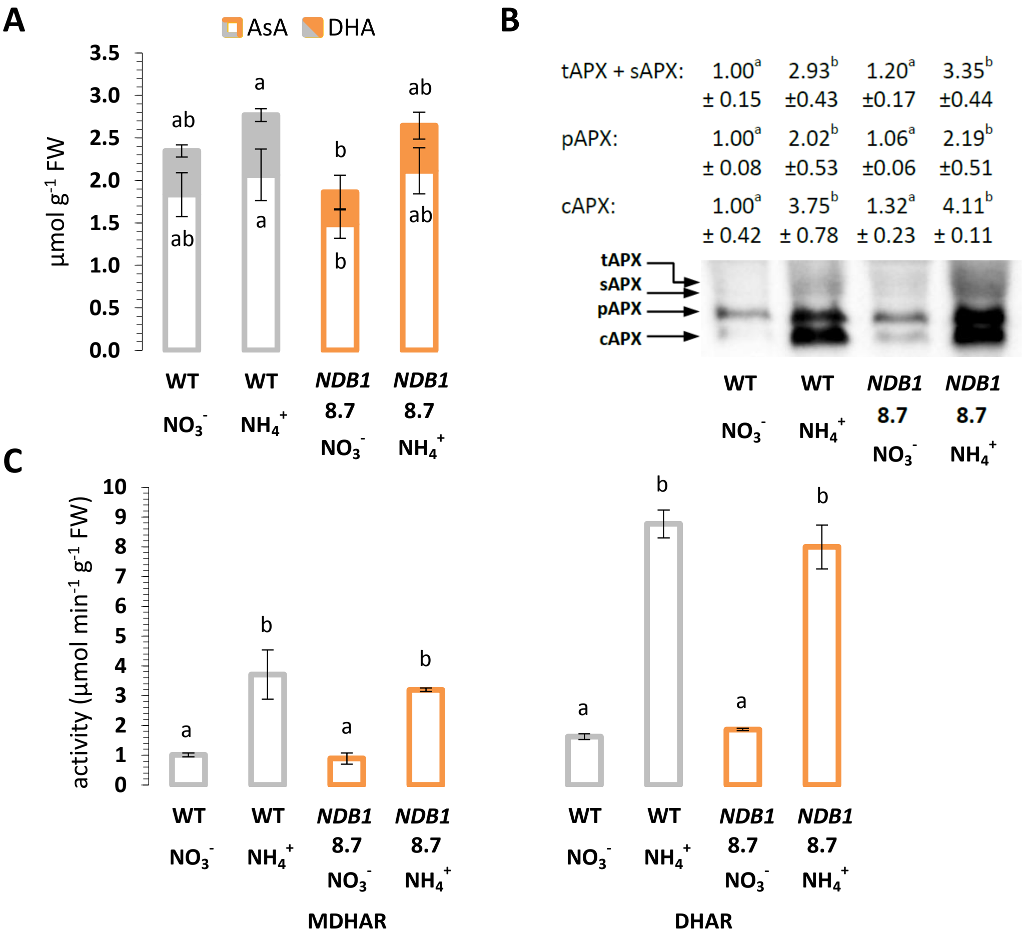

Figure S5. Ascorbate content and ascorbate-related enzymes in *NDB1*-suppressing line 8.7 and WT plants growing on NH_4^+ and NO_3^- (control) as the only source of nitrogen. (A) Concentration of reduced (AsA) and oxidized (DHA) ascorbate and derived AsA/DHA ratio. (B) Ascorbate peroxidase (APX) protein levels. Thylakoid (tAPX, 38 kDa), stromal (sAPX, 33 kDa), peroxisomal (pAPX, 31 kDa), and cytoplasmic (cAPX, 25 kDa) forms of ascorbate peroxidases. (C) Monodehydroascorbate reductase (MDHAR) and dehydroascorbate reductase (DHAR) activities. Bars or bands with different letters are statistically different (P < 0.05) by ANOVA.

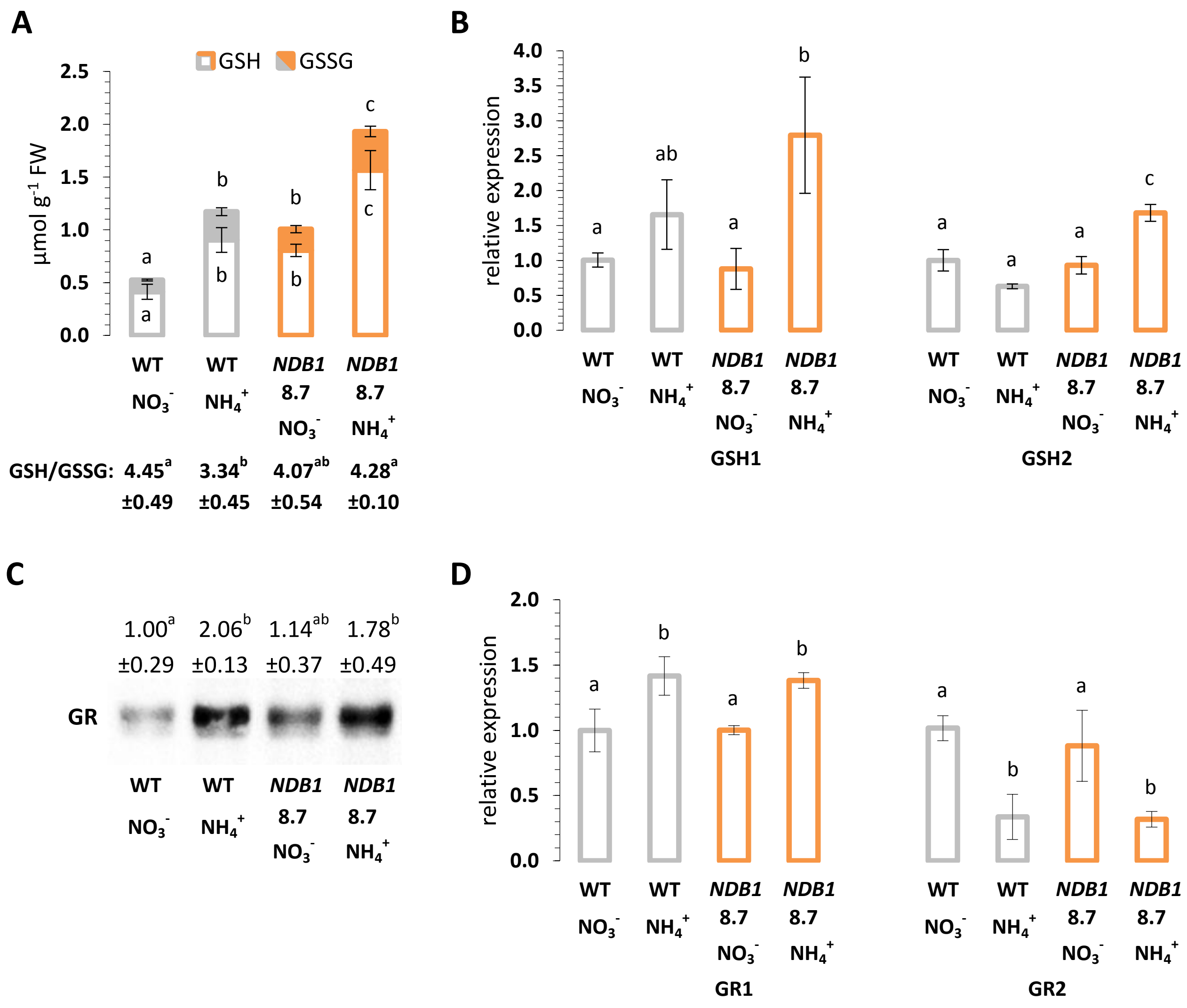

Figure S6. Glutathione content and glutathione metabolism-related enzymes in *NDB1*-suppressing line 8.7 and WT plants growing on NH_4^+ and NO_3^- (control) as the only source of nitrogen. (A) Concentration of reduced (GSH) and oxidized (GSSG) glutathione and derived GSH/GSSG ratio. (B) Transcript levels for *GSH1* and *GSH2*. (C) GR protein levels. (D) Transcript levels for GR isoforms, *GR1* and *GR2*. Bars or bands with different letters are statistically different ($P < 0.05$) by ANOVA.

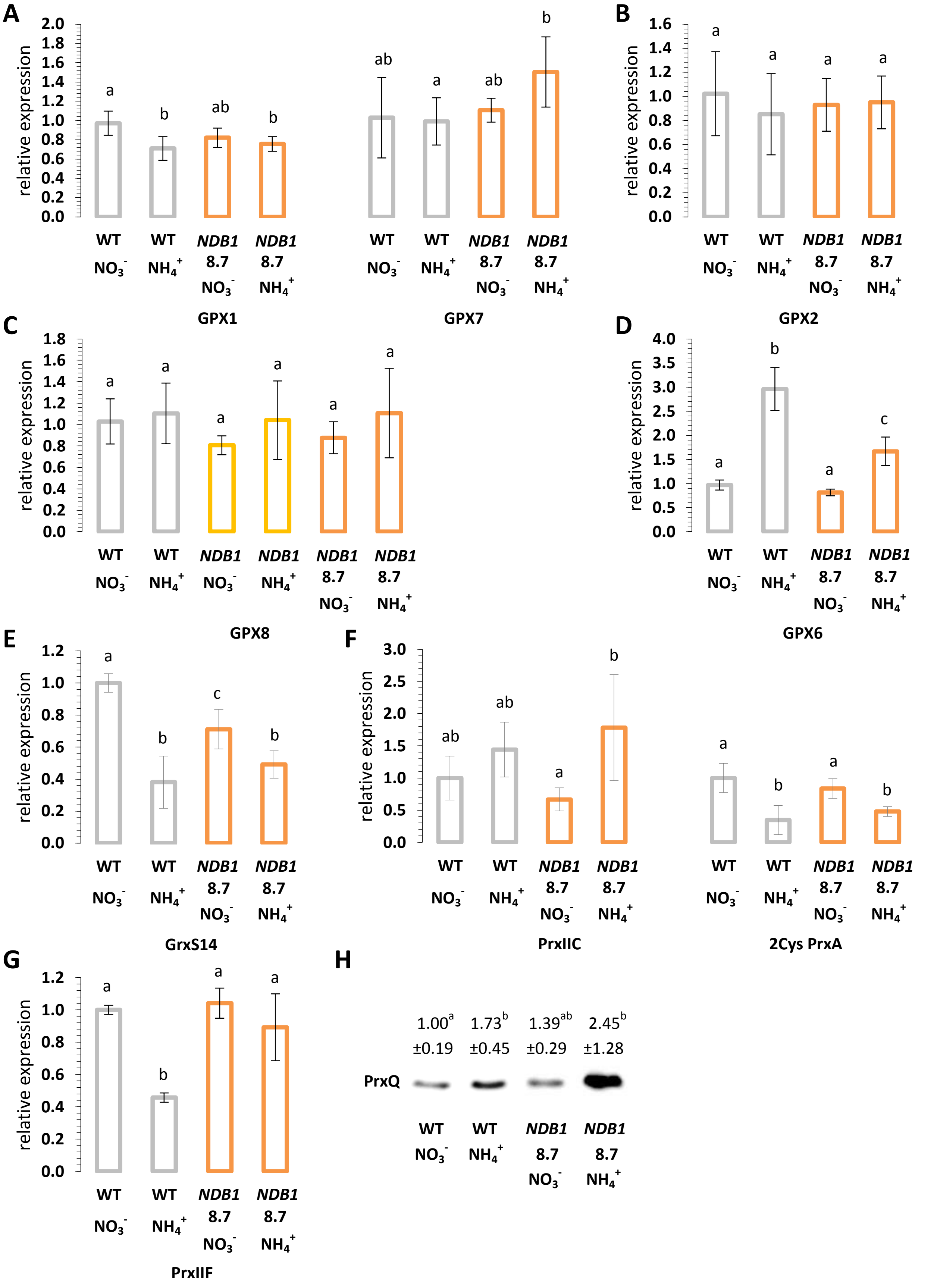

Figure S7. Redox sensors and transmitters in *NDB1*-suppressing line 1.5 (*NDB1*, yellow bars), line 8.7 (*NDB1* 8.7, orange bars), and WT (grey bars) plants growing on NH_4^+ and NO_3^- (control) as the only source of nitrogen. Transcript levels for (A) chloroplast glutathione peroxidase-like (GPX) *GPX1* and *GPX7*, (B) cytosolic *GPX2 and* (C) *GPX8*, (D) mitochondrial *GPX6*, (E) chloroplast glutaredoxin (GRX) *GrxS14*, (F) cytosolic *PrxIIc* and chloroplast *2Cys PrxA*, (G) mitochondrial *PrxIIF*, and (H) chloroplast PrxQ protein levels. Bars or bands with different letters are statistically different ($P < 0.05$) by ANOVA.

Table S1. List of qRT-PCR primers.

Gene	AGI identification	Forward Primer	Reverse Primer
GR2	At3g54660	5′– GATTGAAGGTCGTGGAAAGGTTAT – 3′	5′– CTGGAATGTCAGGAATGAAAGGA – 3′
GPX2	At2g31570	5′– CAAATGTGGTCTGACGGATGC – 3′	5′– CCTAAGAACTGATTACACGGGAA – 3′
GPX7	At4g31870	5′– ATACAAGAACCAAGGATTTGAGATT – 3′	5′– ACCAAGGAAACCACCAGCAT – 3′
GPX8	At1g63460	5′– AATGTGGGATGACAAACTCAAACTAC – 3′	5′– CCAAACTGGTTACAAGGAAATGCT – 3′
GSH1	AT4G23100	5′– GTGGTCAGAACAGGAGTTACGC – 3′	5′– AACACGGATCTACGCTTTGT – 3′
GSH2	At5g27380	5′– CAAAAGTTATCAGAAATCAGGAAATGT – 3′	5′– GGTGTAAACCCAAACGAATGTC – 3′
GrxS14	At3g54900	5′– GCACTCGTCGTCCATAACCA – 3′	5′– TGCCTCAACATCTCATTCTCCA – 3′
2-Cys PrxA	At3g11630	5′– GCCCAACAGAGATTACTGCCTT – 3′	5′– ACTCCGAACGACTTTGAGATTGA – 3′
PrxIIC	At1G65970	5′– CGCACTCAAGTCAAACAAAACT – 3′	5′– GCACCAGGAACACCAAAGAGA – 3′
PrxIIF	At3G06050	5′– GGTCTTCCTGGGGCTTACA – 3′	5′– GACACAGATGACAGAATCAATGC – 3′
TRXx	At1g50320	5′– CTAACCGCCACCTGCTTTC – 3′	5′– TTCCGCCGCATCTTATCAC – 3′
TRXy2	At1g43560	5′– TCACTGTAAGAGCAGCAAAGAAG – 3′	5′– ATAAGATGAATGTGGGCAAAGC – 3′
NTRC	At2g41680	5′– AGAAAATGAGAAAGCAAGCAGAG – 3′	5′– AGGAGCAGTTGTGACACTAAGAGA – 3′
